# The good and the ugly: ANP antagonizes the deleterious effects of aldosterone in hypertensive cardiac remodeling

**DOI:** 10.1186/2050-6511-14-S1-P46

**Published:** 2013-08-29

**Authors:** Hitoshi Nakagawa, Katharina Völker, Heike Oberwinkler, Birgit Gaßner, Charlotte Dienesch, Sandra Umbenhauer, Hideo A Baba, Stefan Frantz, Michaela Kuhn

**Affiliations:** 1 Institute of Physiology, University of Würzburg, Germany; 2 Department of Internal Medicine 1, University Hospital Würzburg, Germany; 3 Department of Pathology and Neuropathology, University Hospital of Essen, University of Duisburg- Essen, Germany

## Background

Atrial natriuretic peptide (ANP), via its guanylyl cyclase A (GC-A) receptor and cyclic GMP formation, exerts cardiac antihypertrophic and antifibrotic actions. Conversely, aldosterone promotes pathological cardiac remodeling via the mineralocorticoid receptor (MR). To investigate whether local cardiac ANP/GC-A signaling counteracts the effects of aldosterone during experimental hypertensive cardiac remodeling.

## Results

We studied the impact of the MR antagonist eplerenone (100 mg/kg/day) on cardiac remodeling after transverse aortic constriction (TAC) in mice with conditional, cardiomyocyte-restricted deletion of GC-A (CM GC-A KO) [1] or cGMP-dependent protein kinase I (CM cGK I KO) [2] and respective controls (n=10 in each group). Left ventricular (LV) hypertrophy and interstitial fibrosis were significantly exacerbated in both CM GC-A KO and cGK I KO mice after TAC. These histological changes were accompanied by decreased LV SERCA2a expression, increased CTGF and LV dilatation together with contractile dysfunction. Eplerenone had no effect on systemic blood pressure but fully prevented these pressure-overload induced cardiac morphological, molecular and functional alterations in both CM GC-A KO and CM cGK I KO mice. However, eplerenone did not inhibit TAC-induced MAPK ERK1/2 phosphorylation/activation, suggesting that the non-genomic effects of aldosterone are not involved in exacerbated hypertensive cardiac remodeling of the KO mice. Intriguingly, in transfected HEK 293 cells, ANP inhibited the aldosterone-induced nuclear translocation of the MR.

## Conclusion

ANP, via GC-A/cGMP/cGKI signaling in cardiac myocytes, attenuates hypertensive cardiac remodeling and dysfunction. These protective ANP effects seem to be mediated at least in part by counterregulation of the deleterious genomic (MR-mediated) cardiac actions of aldosterone.
